# Self-rated health and health-related quality of life among Chinese residents, China, 2010

**DOI:** 10.1186/s12955-016-0409-7

**Published:** 2016-01-12

**Authors:** Wen-lan Dong, Yi-chong Li, Zhuo-qun Wang, Ying-ying Jiang, Fan Mao, Li Qi, Jian-qun Dong, Li-min Wang

**Affiliations:** National Center for Chronic and Noncommunicable Disease Control and Prevention, Beijing, 100050 People’s Republic of China; Chinese Field Epidemiology Training Program, Beijing, 100050 People’s Republic of China

**Keywords:** Self-rated health, Health-related quality of life, Public health

## Abstract

**Background:**

Self-rated health (SRH) and health-related quality of life (HRQOL) are two outcome measures used to assess health status. However, little is known about population-based SRH and HRQOL in China.

**Methods:**

Data from the 2010 China Chronic Disease and Risk Factor Surveillance, a nationally representative sample of 98,658 adults (≥18-year-old) residing in China, were analyzed. SRH was assessed by asking "Would you say that, in general, your health is very good, good, general, poor, or very poor?” HRQOL was assessed by asking “For about how many days during the past 30 days was your health not good due to physical illnesses, injuries, or mental unhealthy?”.

**Results:**

Overall, 6.3 % of participants rated their health as poor or very poor. The prevalence of poor/very poor health increased with advancing age ranging from 2.0 % in the 18–24 year-olds to 14.9 % in those ≥75 years-old, while it decreased with education levels from 13.0 % in illiterates/those with some primary school education to 2.2 % in college graduates or above. Additionally, women were more likely than men to rate their health as poor or very poor (7.2 % vs. 5.4 %). The reported rate of poor/very poor health was higher in western region residents compared to those in the east (7.4 % vs. 5.3 %). The mean numbers of self-reported physically unhealthy days, injury-caused unhealthy days, or mentally unhealthy days during the past 30 days were 1.48, 0.20, and 0.54, respectively. Older adults had more physically unhealthy days than the younger ones ranging from 2.92 days in those ≥ 75 year-old to 0.95 days in 18–24 year-olds. Women had more physically unhealthy days and mentally unhealthy days than men (1.72 vs. 1.23; 0.62 vs. 0.46, respectively). The highest mean number of physically unhealthy days (2.32) was reported by illiterates or those with some primary school education. The highest mean number of mentally unhealthy days (0.86) reported by college graduates or above.

**Conclusions:**

Substantial variations existed in SRH and HRQOL among age groups, gender groups, education groups, and across regions in China. Considering these disparities will be important when developing health policies and allocating resources.

## Background

The World Health Organization (WHO) has defined healthiness as “a state of complete physical, mental, and social well-being and not merely the absence of disease or infirmity” [1]. Health is seen by the public health community as a multidimensional construct that includes physical, mental, and social domains [2]. Self-rated health (SRH) and health-related quality of life (HRQOL) are two outcome measures used to assess health status. These two measures are self-reported, inexpensive, and easy to use.

SRH is easily measured in large population surveys, and is a useful “opener” in interview situations that allow interviewers to seek more nuanced and complex responses about people’s perceptions of their health [3]. Poor SRH status has also been shown to be independently predictive of subsequent morbidity and higher health care utilization [4, 5]. The concept of health-related quality of life is that an individual’s or group’s perceived physical and mental health over time [6]. On the individual level, HRQOL includes physical and mental health perceptions and their correlates, including health risks and conditions, functional status, social support, and socioeconomic status [7–9]. On the community level, HRQOL includes resources, conditions, policies, and practices that influence a population’s health perceptions and functional status [10]. HRQOL questionnaires on perceived physical and mental health and function have become an important component of health surveillance and are generally considered valid indicators of service needs and intervention outcomes [6].

Measuring SRH and HRQOL through continual surveillance would identify health disparities, track population trends, and provide the public’s perspective to guide health policies [6, 11]. SRH and HRQOL have been tracked in many countries. For instance, in the early 1990s, the Centers for Disease Control and Prevention (CDC) in the U.S. developed and validated a brief set of questions to track SRH and HRQOL in states and communities. From 1993 through 2001, more than 1.2 million adults aged 18 years old or above in the U.S. answered these questions on the population-based Behavioral Risk Factor Surveillance survey (BRFSS) [6, 11]. SRH and HRQOL have also been being tracked through National Health Survey (NHS) in Candia, Australian, French, Xincarpor, et al. [3, 12–14].

However, there is limited information regarding the SRH and HRQOL status in China. The aim of this study was to estimate status of SRH and HRQOL for Chinese adults based on the nationally-representative data from China Chronic Disease and Risk Factor Surveillance (CCDRFS) in 2010. We hypothesize that both SRH and HRQOL will differ among subgroups such as age, gender, education, marital status, residence location (urban/rural), and geographic location groups. Based on the findings from this study, we discussed the implications of the SHR and HRQOL on developing health policies in China.

## Methods

### Surveillance and study sample

The CCDRFS is an ongoing, nationally representative surveillance survey administered by China’s National Center for Chronic and Noncommunicable Disease Control and Prevention. The 2010 CCDRFS was carried out from August to November in 2010 using the national disease surveillance points system, which encompassed 162 districts/counties from all 31 provinces, autonomous regions and municipalities in the mainland China. The establishment, history, and degree of representativeness of the national disease surveillance points system were explained elsewhere [15, 16].

The 2010 CCDRFS was approved by the ethics committee of the Chinese Center for Disease Control and Prevention and the written informed consent forms were obtained from each participant before data collection. The 2010 CCDRFS was conducted by gathering participants in certain central locations. The data were collected in face-to-face interviews on SRH and HRQOL questionnaires. All investigators participated in national or provincial training courses and were qualified to engage in field activities after passing standard exams. Detailed information on quality control and the data analyses in the 2010 CCDRFS has been published elsewhere [17].

Multistage stratified cluster sampling was used to select participants for the 2010 CCDRFS. In the first stage of sampling, four townships were randomly sampled from each surveillance district/county using the method of probability proportional to size. Three villages or residential areas were then sampled from each chosen township using the same method as in the previous stage. Subsequently, a residential group (at least 50 families) was sampled from each chosen village or residential area by simple random sampling. Finally, an individual of at least 18 years old was sampled in each family by means of a Kish grid. About 9.4% of the sampled families could not be located after three attempts and these households were replaced by others with similar family structures. A total of 98658 interviews were included in the final analyses [18].

### Measures

SRH and HRQOL were assessed based on the participants’ answers for the following four questions:

1. Would you say that, in general, your health is very good, good, general (not good/not poor), poor, or very poor? [SRH]
2. Now thinking about your physical illness, for how many days during the past 30 days was your health not good due to physical illness? [Physically unhealthy days]
3. Now thinking about your injury, which includes accident injury or intentional damage such as fall, traffic, etc., for how many days during the past 30 days was your health not good due to injury? [Injury-caused unhealthy days]
4. Now thinking about your mental health, which includes stress, depression, and problems with emotions, for how many days during the past 30 days was your health not good due to mental unhealthy? [Mentally unhealthy days]

We obtained data based on demographic characteristics (age, gender, and marital status), socioeconomic status (educational level), residence location (rural/urban), and geographic region of China. The corresponding subcategories were shown in Table 1. We calculated 1) the percentages and 95 % confidence interval [CI] for respondents reporting very good, good, general, poor, and very poor based on the SRH questions and 2) the mean number and 95 % CI for the physically unhealthy days, injury-caused unhealthy days, and mentally unhealthy days based on HRQOL questions [19].

**Table 1:** Characteristics of the study sample of the 2010 CCDRFS survey

Characteristic	Respondents (*n* = 98658)		
	No.	(%)	Weighted ^a^ %
Age group (years)			
18-24	8372	8.5	16.1
25-34	13534	13.7	18.2
35-44	23271	23.6	23.3
45-54	22837	23.2	18.2
55-64	18362	18.6	13.6
65-74	8902	9.0	7.0
≥75	3380	3.4	3.6
Gender			
Man	45143	45.8	50.8
Woman	53515	54.2	49.2
Education			
Illiterate or some primary school	24108	24.4	19.5
Primary school graduate or some junior high school	18965	19.2	18.2
Junior high school graduate or some senior high school	31378	31.8	35.8
Senior high school graduate or some college	16045	16.3	17.5
College graduate or above	8162	8.3	9.0
Marital status			
Single	8432	8.5	8.3
Married or cohabiting	80193	81.3	78.0
Separated/divorced/widowed/others	10033	10.2	13.7
Place of residence			
Urban	38928	39.5	31.2
Rural	59730	60.5	68.8
Geographic location			
Eastern China	32965	33.4	39.9
Central China	30569	31.0	32.5
Western China	35124	35.6	27.6
Total	98658	100.0	100.0

^a^ Complex weights were used to obtain nationally representative estimates

Source: Data from the 2010 China Chronic Disease and Risk Factor Surveillance survey (CCDRFS)

### Statistical analysis

In the present study, weighting was conducted in all statistical analyses to obtain nationally representative estimates. The weights were the products of sampling selection weight, which was the reciprocal of the probability of a particular individual being selected, and a post-stratification factor that adjusted for age, rural/urban residence, and geographic location in accordance with the 2009 Chinese population estimates obtained from the National Bureau of Statistics of China [20].

We first determined the sample characteristics and then estimated the percent of SRH and the mean number of physically, injury-caused, and mentally unhealthy days on the basis of a specific characteristic of population, such as age, gender, marital status, education, rural/urban residence, and geographic location. Age was divided into 7 groups: 18–24, 25–34, 35–44, 45–54, 55–64, 65–74, and 75+. Marital status was classified into 3 categories: single, married/cohabiting, and separated/divorced/widowed/others. Socioeconomic status was assessed based on the education levels that was categorized as 5 groups: illiterate or some primary school, primary school graduate or some junior high school, junior high school graduate or some senior high school, senior high school graduate or some college, and college graduate or above. The country was divided by geography: eastern, central, and western regions according to the National Bureau of Statistics. The eastern region includes Beijing, Tianjin, Hebei, Liaoning, Shanghai, Jiangsu, Zhejiang, Fujian, Shandong, Guangdong, and Hainan. The central region includes Shanxi, Jilin, Heilongjiang, Anhui, Jiangxi, Henan, Hubei, and Hunan. The western region includes Inner Mongolia, Guangxi, Chongqing, Sichuan, Guizhou, Yunnan, Tibet, Shaanxi, Gansu, Qinghai, Ningxia, and Xinjiang.

In Table 2, We combined the categories poor and very poor because the percentage of very poor is very low and conducted Rao-Scott χ2 tests for percentages of SRH (very good, good, general, and poor/very poor) to test the differences in proportion by categories.

**Table 2:** Weight percent of self-rated health among Chinese adults aged ≥18 years, China, 2010 (*n* = 98658 missing = 20)

Characteristic		Very good		Good		General		Poor/Very poor	*p*
	Number of respondents	Weighted percent (95 % CI)	Number of respondents	Weighted percent (95 % CI)	Number of respondents	Weighted percent (95 % CI)	Number of respondents	Weighted percent (95 % CI)	
Age group (years)									
18-24	1405	18.5 (15.5-21.5)	4693	55.4 (52.2-58.6)	2109	24.1 (21.1-27.1)	165	2.0 (1.6-2.5)	*P*<0.01
25-34	1546	13.1 (11.2-15.1)	7633	55.1 (52.7-57.4)	3967	29.1 (26.6-31.5)	387	2.7 (2.3-3.3)	
35-44	1805	9.4 (7.9-11.0)	12205	51.4 (49.1-53.7)	8092	34.4 (32.3-36.5)	1165	4.8 (4.2-5.5)	
45-54	1146	6.3 (5.1-7.5)	10603	45.5 (43.7-47.4)	9272	40.5 (38.4-42.6)	1809	7.7 (7.0-8.4)	
55-64	698	4.4 (3.6-5.3)	7274	38.8 (36.8-40.8)	8426	46.1 (44.0-48.2)	1961	10.6 (9.7-11.7)	
65-74	228	3.0 (2.3-3.6)	3028	33.5 (31.6-35.3)	4410	49.3 (47.8-50.9)	1232	14.2 (12.7-16.0)	
≥75	92	2.9 (2.1-3.8)	1033	30.0 (27.7-32.3)	1766	52.2 (48.9-55.4)	488	14.9 (12.6-17.4)	
Gender									
Man	3737	11.1 (9.6-12.6)	22136	49.4 (47.5-51.2)	16437	34.1 (32.4-35.8)	2827	5.4 (4.9-6.0)	*P*<0.01
Woman	3183	8.1 (6.9-9.4)	24333	46.3 (44.6-48.1)	21605	38.3 (36.6-40.0)	4380	7.2 (6.5-8.0)	
Education									
Illiterate or some primary school	813	4.2 (3.4-5.2)	9962	39.3 (37.0-41.6)	10269	43.5 (41.5-45.6)	3054	13.0 (11.7-14.4)	*P*<0.01
Primary school graduate /some junior high school	1021	6.6 (5.2-8.4)	8839	46.6 (44.2-49.1)	7512	38.9 (36.4-41.4)	1590	7.9 (7.1-8.8)	
Junior high school graduate/some senior high school	2765	11.6 (9.8-13.8)	15849	51.4 (48.9-53.9)	11121	32.8 (30.6-35.1)	1637	4.2 (3.7-4.8)	
Senior high school graduate/some college	1417	12.3 (10.7-13.9)	7814	50.1 (48.0-52.2)	6111	34.0 (31.6-36.4)	703	3.7 (3.2-4.2)	
College graduate or above	904	14.5 (12.7-16.5)	4005	50.8 (48.5-53.1)	3029	32.5 (29.6-35.6)	223	2.2 (1.8-2.7)	
Marital status									
Single	1323	18.2 15.7-21.03)	4510	53.6(50.5-56.6)	2307	25.4(22.5-28.4)	291	2.9(2.3-3.6)	*P*<0.01
Married or cohabiting	5247	8.7(7.4-10.2)	37903	47.7(45.9-49.5)	31259	37.3(35.5-39.2)	5767	6.3 5.7-7.0)	
Separated/divorced/widowed/others	350	4.3(3.5-5.3)	4056	40.5(36.9-44.1)	4476	43.3(40.5-46.2)	1149	11.8(10.3-13.6)	
Place of residence									
Urban	3029	9.8 (8.5-11.1)	17371	45.2 (42.8-47.6)	16129	39.3 (36.8-41.8)	2392	5.7 (4.7-6.8)	*P*<0.05
Rural	3891	9.6 (7.7-11.4)	29098	49.1 (46.9-51.3)	21913	34.7 (32.6-36.9)	4815	6.6 (5.9-7.4)	
Geographic location									
Eastern China	3137	13.0 (10.8-15.1)	14938	47.1 (44.6-49.6)	12748	34.6 (32.3-36.9)	2134	5.3 (4.5-6.2)	*P*<0.01
Central China	1877	7.3 (6.1-8.4)	14185	48.4 (44.8-51.9)	12130	37.8 (34.5-41.0)	2376	6.6 (5.6-7.8)	
Western China	1906	7.6 (4.2-10.9)	17346	48.5 (45.6-51.4)	13164	36.5 (33.2-39.8)	2697	7.4 (6.3-8.7)	
Total	6920	9.7 (8.3-10.9)	46469	47.9 (46.2-49.6)	38042	36.2 (34.5-37.8)	7207	6.3 (5.7-6.9)	

Note: Percents were weighted to represent the total population of the national disease surveillance points system with post-stratification for age and gender.

The number in parentheses is the percent of the 95% confidence interval which taking into account the complex survey design.

Source: Data from the 2010 China Chronic Disease and Risk Factor Surveillance survey

In Table 3, we used CIs to compare the mean number of physically unhealthy days, injury-caused unhealthy days, and mentally unhealthy days by categories. If the 95 % CIs of these estimates did not overlap, these estimates were considered different from each other at the α = 0.05 significance level [21, 22].

**Table 3:** Mean number unhealthy days during the past 30 days among Chinese adults aged ≥ 18 years, China, 2010 (*n* = 98658)

Characteristic	Physically unhealthy days ^a^		Injury-caused unhealthy days ^b^		Mentally unhealthy days ^c^	
	Number of respondents	Mean number of days ( 95 % CI )	Number of respondents	Mean number of days ( 95 % CI	Number of respondents	Mean number of days ( 95 % CI
Age group (years)						
18-24	7530	0.95 ( 0.79-1.10 )	7548	0.26 ( 0.17-0.35 )	7553	0.58 ( 0.45-0.71 )
25-34	12106	0.93 ( 0.81-1.05 )	12109	0.20 ( 0.13-0.26 )	12074	0.55 ( 0.46-0.65 )
35-44	21058	1.15 ( 1.04-1.27 )	21079	0.15 ( 0.11-0.19 )	21009	0.50 ( 0.43-0.57 )
45-54	20522	1.70 ( 1.51-1.88 )	20700	0.23 ( 0.19-0.27 )	20607	0.57 ( 0.48-0.65 )
55-64	16385	2.12 ( 1.93-2.31 )	16628	0.22 ( 0.18-0.26 )	16545	0.56 ( 0.49-0.63 )
65-74	7813	2.74 (2.48-3.01 )	7999	0.20 ( 0.14-0.26 )	7949	0.49 ( 0.40-0.57 )
≥75	2861	2.92 (2.42-3.43 )	2948	0.21 ( 0.13-0.28 )	2930	0.41 ( 0.31-0.51 )
Gender						
Man	40279	1.23 ( 1.12-1.34 )	40572	0.23 ( 0.18-0.28 )	40481	0.46 ( 0.40-0.53 )
Woman	47996	1.72 ( 1.56-1.88 )	48439	0.17 ( 0.14-0.21 )	48186	0.62 ( 0.54-0.70 )
Education						
Illiterate or some primary school	7404	2.32 ( 2.12-2.52 )	7476	0.22 ( 0.17-0.27 )	7465	0.50 ( 0.43-0.58 )
Primary school graduate/some junior high school	14658	1.66 ( 1.47-1.84 )	14773	0.24 ( 0.15-0.32 )	14754	0.51 ( 0.41-0.60 )
Junior high school graduate/some senior high school	28476	1.12 (1.00-1.23 )	28652	0.21 ( 0.16-0.25 )	28605	0.45 ( 0.37-0.53 )
Senior high school graduate/some college	16851	1.27 ( 1.09-1.46 )	17025	0.19 ( 0.14-0.25 )	16938	0.62 ( 0.53-0.72 )
College graduate or above	20886	1.17 ( 0.99-1.34 )	21085	0.13 ( 0.09-0.18 )	20905	0.86 ( 0.72-1.00 )
Marital status						
Single	7618	1.07 ( 0.93-1.21 )	7624	0.26 ( 0.16-0.36 )	7629	0.65 ( 0.52-0.78 )
Married or cohabiting	71647	1.47 ( 1.34-1.59 )	72233	0.20 ( 0.16-0.24 )	71923	0.51 ( 0.44-0.58 )
Separated/divorced/widowed/others	9010	2.24 ( 1.90-2.57 )	9154	0.20 ( 0.14-0.27 )	9115	0.66 ( 0.53-0.78 )
Place of residence						
Urban	35561	1.57 ( 1.37-1.78 )	36182	0.16 ( 0.14-0.19 )	36093	0.65 ( 0.55-0.74 )
Rural	52714	1.43 ( 1.27-1.59 )	52829	0.22 ( 0.17-0.28 )	52574	0.49 ( 0.40-0.58 )
Geographic location						
Eastern China	29155	1.43 ( 1.23-1.63 )	29398	0.18 ( 0.14-0.21 )	29341	0.51 ( 0.40-0.61 )
Central China	28048	1.30 ( 1.08-1.51 )	28444	0.16 ( 0.11-0.21 )	28247	0.49 ( 0.37-0.61 )
Western China	31072	1.76 ( 1.50-2.01 )	31169	0.30 ( 0.18-0.42 )	31079	0.64 ( 0.49-0.79 )
Total	88275	1.48 ( 1.35-1.60 )	89011	0.20 ( 0.17-0.24 )	88667	0.54 ( 0.47-0.61 )

^a^ Number of respondents to physically unhealthy day = 88275, missing =10383

^b^ Number of respondents to injury-caused unhealthy days =89011, missing = 9647

^c^ Number of respondents to mental unhealthy = 88667, missing = 9991

Note: The mean numbers of days were weighted to represent the total population of the national disease surveillance points system with post-stratification for age and gender;The number in parentheses is the mean number of days of the 95% confidence interval which taking into account the complex survey design.

Source: Data from the 2010 China Chronic Disease and Risk Factor Surveillance survey

In Table 4, we examined 1) the independent effects of covariates on SRH (very good, good, general, poor/very poor) by modeling a multiple ordered logistic regression and 2) the independent effects of covariates on unhealthy days by multiple liner regression. The age, gender, marital status, education, rural/urban residence, and geographic location were independent variables. Logistic model was presented as odds ratios (OR) with 95 % CI and liner regression model was presented as β with *P* value. These ordered categorical variables were tested as a continuous variable in a logistic regression model [23]. The respondents who reported "don't know/not sure" or "refused" were excluded from the analysis. All statistical analysis were performed using SAS version 9.3 (SAS Institute Inc., Cary, USA) and CIs were estimated while accounting for complex sample design using the Taylor’s series method [24] with finite population correction.

**Table 4:** Independent effects of covariates on self-rated health and unhealthy days in individuals, China, 2010

	Self-rated health ^a^	Physically unhealthy days ^d^		Injury-caused unhealthy days ^d^		Mentally unhealthy days ^d^	
Characteristic Cumulative OR ^b^ (95 % CI ^a^)	Cumulative OR ^b^ (95 % CI ^c^)	*β*	*P*	*β*	*P*	*β*	*P*
Age group (years)							
18-24	1.00	0.00	.	0.00	.	0.00	.
25-34	1.36 (1.22-1.50)	0.04	0.63	−0.06	0.24	−0.03	0.63
35-44	1.83 (1.62-2.07)	0.32	<0.01	−0.08	0.26	0.02	0.82
45-54	2.71 (2.37-3.10)	0.81	<0.01	−0.01	0.86	0.10	0.21
55-64	3.58(3.10-4.13)	1.17	<0.01	−0.01	0.85	0.11	0.20
65-74	4.62 (3.93-5.44)	1.76	<0.01	−0.03	0.68	0.07	0.47
≥75	4.88 (4.05-5.88)	1.78	<0.01	−0.03	0.73	−0.06	0.49
Gender							
Man	1.00	0.00	.	0.00	.	0.00	.
Woman	1.25(1.19-1.31)	0.47	<0.01	−0.03	0.06	0.22	<0.01
Education							
Illiterate or some primary school	1.00	0.00	.	0.00	.	0.00	.
Primary school graduate/some junior high school	0.89(0.81-0.96)	−0.19	0.04	0.01	0.79	0.07	0.15
Junior high school graduate/some senior high school	0.73 (0.66-0.80)	−0.43	<0.01	−0.03	0.12	−0.00	0.90
Senior high school graduate/some college	0.73 (0.64-0.83)	−0.29	0.01	−0.06	0.03	0.17	<0.01
College graduate or above	0.68 (0.56-0.82)	−0.24	0.04	−0.05	0.07	0.45	<0.01
Marital status							
Single	0.94 (0.83-1.06)	0.14	0.22	0.02	0.73	−0.02	0.83
Married or cohabiting	0.93 (0.85-1.04)	−0.03	0.73	0.00	0.93	−0.16	<0.01
Separated/divorced/widowed/others	1.00	0.00	.	0.00	.	0.00	.
Place of residence							
Urban	1.15 (0.96-1.37)	0.12	0.38	−0.03	0.27	0.07	0.33
Rural	1.00	0.00	.	0.00	.	0.00	.
Geographic location							
Eastern China	1.00	0.00		0.00		0.00	
Central China	1.42 (1.15-1.75)	−0.05	0.70	−0.03	0.07	−0.00	0.93
Western China	1.37 (1.07-1.75)	0.32	0.03	0.11	0.12	0.19	0.07

^a^ Examined the independent effects of covariates on SRH (very good, good, general, poor/very poor) by modeling a multiple ordered logistic regression

^b^ Cumulative OR from an ordinal logistic regression model with adjustment for all covariates. Self-rated health (very good/good/ general/poor/very poor) was the dependent variable. Each OR reflects the cumulative odds of rating poorer health versus better health against the cumulative odds in the reference group. Hence, the cumulative OR represents the average effect of the covariate on the cumulative odds of rating poorer health.

^c^ The 95 % CIs take into account the complex survey design. It was no significantly difference between the covariate group and the reference group if 1 fall within 95 % CI OR, and vice versa.

^d^ Examined the independent effects of covariates on unhealthy days by multiple liner regression.

Source: Data from the 2010 China Chronic Disease and Risk Factor Surveillance survey

## Results

### Characteristics of the respondents

The sample characteristics are shown in Table 1. The majority of the respondents were between 35 and 54 years old (46.8 %). There were more women than men (54.2 % vs. 45.8 %). About one third (31.8 %) were junior high school graduates or some high school. 81.3 % were either married or cohabiting. 60.5 % lived in rural areas and 35.6 % lived in western China. The Table 1 also shows the distributions of the various characteristics after weighting for obtaining nationally representative estimates.

### Distribution of SRH

Table 2 shows the distribution of SRH among Chinese adults aged ≥18 years in 2010. Overall, 9.7 % rated their health as being very good, 47.9 % as good, 36.2 % as general, and only 6.3 % as poor or very poor. The proportion of SRH was significantly different among age groups (χ^2^ = 21.07, *P* < 0.01), gender groups (χ^2^ = 60.17, *P* < 0.01), education groups (χ^2^ = 68.03, *P* < 0.01), marital status groups (χ^2^ = 72.14, *P* < 0.01), urban–rural group (χ^2^ = 2.98, *P* < 0.05), and geographic location (χ^2^ = 4.35, *P* < 0.01).

### Distribution of HRQOL

Table 3 shows the mean number of physically unhealthy days, injury-caused unhealthy days, and mentally unhealthy days during the past 30 days among Chinese adults aged ≥ 18 years.

#### Physically unhealthy days

Adults reported that they had an average of 1.48 physically unhealthy days during the past 30 days. The physically unhealthy days increased modestly with advancing age ranging from 0.95 days in 18–24 years old age group to 2.92 days in 75 years old age group. Woman reported more physically unhealthy days than man (1.72 vs. 1.23). The adults with less education reported more physically unhealthy days compared to those with more education ranging from 2.32 days with illiterate or some primary school to 1.17 days with college graduate or above. The highest physically unhealthy days were reported in the adults who were separated, divorced, widowed, or in other marital status groups (2.24 days).

#### Injury-caused unhealthy days

Adults reported that they had an average of 0.20 (95 % CI: 0.17-0.24) injury-caused days during the past 30 days. The differences of mean injury days were not found to be significant among age groups, gender groups, education groups, marital status groups, urban–rural groups, and geographic location groups.

#### Mentally unhealthy days

Adults reported that they suffered an average number of 0.54 mentally unhealthy days during the past 30 days. Women reported more mentally unhealthy days than men (0.62 vs. 0.46). The adults with more education reported more mentally unhealthy days than those with less education. The mentally unhealthy days decreased from 0.86 days with college graduates or above to 0.50 days with illiterate or some primary school. There were no significant differences of mentally unhealthy days among age groups, marital status groups, urban–rural groups, and geographic location groups.

#### Associated factors with SRH and HRQOL

Table 4 shows the independent effect of various covariates on the SRH by ordinal logistic regression and unhealthy days by liner regression at the individual level as indicated.

Age, gender, education, and geographic location were independently associated with the SRH. For instance, in adults aged 75 years or older, the cumulative odds of reporting the poorer health 4.88 times higher than those in 18–24 years old. The cumulative odds increased steadily with increasing age and declined steadily with educational levels. Women were 1.25 times more likely to rate poorer health, than men. The adults in the central region were 1.42 times and in the western region were 1.37 times higher than those in the eastern region.

Age, gender, education, and geographic location were independently associated with the physical unhealthy days. For instance, in adults aged 75 years or older, reporting unhealthy days increase 1.78 days than those in 18–24 years old. β value increased steadily with increasing age and declined steadily with educational levels. Women reported more physically unhealthy days (0.47) than man. Adults in the west region also reported more physically unhealthy days (0.32) than those in the eastern region.

Gender and education were independently associated with the mentally unhealthy days. Women reported more mentally unhealthy days (1.37) than man. Adults with senior high school graduate and college graduate reported 0.17 and 0.45 more mentally unhealthy days than those with illiterate or some primary school, respectively.

## Discussion

We used a large and nationally representative sample to examine for the first time the distribution of SRH and HRQOL status among Chinese adults in 2010. We found that the proportion of SRH was significantly different among age groups, gender groups, education groups and geographic location groups. Adults who were older, woman, lived in the western region, or had less education were more likely to report poorer SRH. Older adults reported more physically unhealthy days than younger ones. Women reported more physically unhealthy days and mentally unhealthy days than men. Adults with more education reported fewer physically unhealthy days and more mentally unhealthy days than those with less education. Adults in the west region reported more physically unhealthy days than those in the eastern region.

### SRH and HRQOL

This study showed that 6.3 % Chinese adults rated their health as poor or very poor, 47.9 % good and 9.7 % very good. Collectively, Chinese adults appear more positive in their ratings of health compared to many other countries.

The reported poor SRH status varied in different countries. Among adults aged 18 years old or above, the estimated overall rate of self-rated fair or poor (excellent, very good, good, fair and poor) health ranged from 10.1 % in Minnesota to 30.9 % in Puerto Rico [25], while 15.9 % adults rated their health as fair or poor in U.S. in 2009 [26]. 9.7 % Canadians reported fair or poor health in 2012 [27]. Approximately15 % of the Australian population rated their health as fair or poor in 2007 [28]. Only 1.5 % Singaporeans rated their health as bad or very bad in 2001 [3]. The differences might be also partially due to the differences of the methodologies used for accessing SRH among different countries, such as the methods involved in eliciting a response, the questions phrased, and the methods used for interviewing.

A study showed the strong and nearly linear relationship between SRH status and the use of physician services during the following year, which mean the use of health services will increase with the SRH as poor or very poor [29]. Therefore, health care providers might use SRH to find persons with high risk of diseases and provide early treatment to reduce the cost of medical care.

We found that the mean number of physically unhealthy days was 1.48, injury-caused unhealthy days 0.20, and mentally unhealthy days 0.54 during the past 30 days in China. Therefore the physical illness remains a major problem for Chinese adults.

HRQOL can be influenced by participants’ experiences, beliefs, expectations, and perceptions [21]. Mean numbers of physically and mentally unhealthy days were 3.6 and 3.5, respectively during the past 30 days in the U.S. in 2009 [22, 30]. Telephone interviews were used to collect data on SRH and HRQOL in BRFSS surveys in the U.S. while face-to-face interviews were conducted by gathering participants in certain central locations for the CCDRFS survey in China. Thus, adults who were severely ill or injured were not able to be interviewed. This may explain the potential reason why fewer physically unhealthy days and mentally unhealthy days were reported in China than those in U.S.

### Age

Our study showed that older adults reported more physically unhealthy days than younger people as well as poorer SRH status. The mean number of physically unhealthy days was almost 3 days during the past 30 days and about 15 % rated their health poor or very poor among those aged 75 and over. The poor health status of older people might be due to the increased chronic diseases. The reports from CCDRFS showed that 66.9 % had hypertension, 19.6 % had diabetes, 12.6 ‰ and 16.8 ‰ self-reported incidences of myocardial infarction or stroke among the elderly aged 60 years or older in 2010 [31]. The population aged 60 and older reached 221 million in 2015, and now consists of 16 % of the total Chinese population [31]. The population aging process will bring a significant impact on disease patterns and health status among Chinese. Our finding further suggests that poor health statuses in the elderly may be one of the main concerns for policy makers. The health care and medical services for the elderly population should be taken into account for implementing policies and interventions.

### Gender

We found that women reported more physically unhealthy days and mentally unhealthy days than men as well as poorer SRH status. Similar findings were also reported in Singapore and the U.S. [3, 32]. The poorer health status of women is speculated to be caused by their physiological features, roles in society, numerous responsibilities, juggling work-life, and family duties. Furthermore, the previous study has shown that depression and anxiety disorders were more common in women than men [33]. Additionally, a U.S. study reported that U.S. women of lower social status were more likely to report poor health [34]. Thus, our finding indicates that more attention is needed for women’s health and appropriate public health interventions should be implemented to improve women’s health statuses in China. In addition, it would be beneficial to women’s health if their social statuses are improved.

### Education

In this study, we found that the physically unhealthy days declined with improving educational levels while mentally unhealthy days increased. Adults with higher education could typically obtain better jobs with higher incomes and better medical care, possibly leading to better physical status. However, adults with higher education might be subjected to higher expectations, possibly pressuring them into more mental health problems.

Our study also showed that the prevalence of self-reporting health as poorer was associated with less education. This finding was consistent with the reports from BRFSS [32], indicating that 1) strengthening national education is good for improving population health status and 2) increasing health education services and health promotion activities might improve the health status of the general Chinese population, and even more so in people with lower education levels.

### Place of residence and geographic location

Our study showed adults in the west region and in the central region reported poorer health than those in the eastern region. The finding indicates that the substantial disparity in health status still exists across regions in China in 2010. SRH encompasses physical health, mental health, and functional capacity of persons [35] and is a proxy indicator for perceived burden of acute and chronic health conditions [6]. The finding of BRFSS in U.S. indicated substantial variations in fair or poor health at the state and local levels suggest differences in the underlying burden of chronic diseases, health-care coverage, and health behaviors among states and territories [25]. Therefore, our findings call for immediate public health intervention to eliminate the disparity across regions. Fortunately, one strategy to eliminate the health disparities across regions was proposed in "Healthy China 2020" Strategy Research Report in 2008 [36], asserting that more health policies and allocating resources for health should be applied to eliminate the health disparities across regions in China.

### Limitations

There are several limitations in this study. First, the cross-sectional design does not allow for any inferences on causality. Second, although the-four-questions measuring SRH and HRQOL had good construct validity, predictive validity, reliability, and responsiveness [6], many respondents appeared to give a response that represented the overall impression of their health over the recent past versus an actual count of days. Therefore the data collected are susceptible to recall bias. Third, all participants were interviewed directly in certain central locations, thus the adults who were severely ill or injured were unable to be surveyed. Nonetheless, the CCDRFS is the largest nationwide Chinese survey and is a useful data source for evaluating the population health status. Thus, this study suggests that appropriate public health interventions are essential for improving health among different groups in the Chinese population.

## Conclusions

Collectively, in this study, we assessed for the first time the status of SRH and HRQOL for adults from all provinces in China. Substantial variation exists in SRH and HRQOL status among age groups, gender groups, education groups, and regions. Taking these disparities into account is extremely important for identifying the health-related needs of vulnerable populations, developing health policy, and allocating resources.

## Abbreviations

SRH: self-rated health
HRQOL: health-related quality of life
CDC: the Centers for Disease Control and Prevention
BRFSS: the population-based Behavioral Risk Factor Surveillance survey
NHS: National Health Survey
CCDRFS: China Chronic Disease and Risk Factor Surveillance
CI: confidence interval
OR: odds ratios
